# Engineering Two Sustainable Packaging Platforms from Agricultural Waste: Microbial Melanin-Enhanced Single-Cell Protein and Bacterial Cellulose Films

**DOI:** 10.3390/foods15193394

**Published:** 2026-09-23

**Authors:** Danai Ioanna Koukoumaki, Freideriki Gala, Eirini Tolia, Dimitris Sarris

**Affiliations:** Laboratory of Physico-Chemical and Biotechnological Valorization of Food By-Products, Department of Food Science and Nutrition, School of the Environment, University of the Aegean, 81400 Lemnos, Greece; danaikouk@aegean.gr (D.I.K.); fnsm223004@fns.aegean.gr (F.G.); fnsm223011@fns.aegean.gr (E.T.)

**Keywords:** microbial melanin, packaging, biopolymers, agricultural wastes

## Abstract

This study proposes a sustainable biorefinery strategy to develop alternative packaging films by integrating three microbial-derived materials produced from agro-industrial residues. Cheese whey and citrus waste were used as renewable substrates for producing single-cell protein (SCP), bacterial cellulose (BC), and microbial melanin (MM). SCP consists of dried microbial biomass rich in protein, while BC is a renewable biopolymer with outstanding physicochemical properties. MM, a natural pigment produced by black yeasts, is a multifunctional biopolymer with considerable technological potential. In this work, SCP and BC were employed as two distinct film-forming matrices, whereas MM was incorporated into both types of films as a functional additive to enhance their performance. To the best of the authors’ knowledge, this is the first study reporting the incorporation of microbial melanin into both SCP- and BC-based films. SCP and BC were produced by *Kluyveromyces marxianus* EXF-5288 and *Komagataeibacter rhaeticus* UNIWA AAK2, respectively, while microbial melanin was produced by *Exophiala phaeomuriformis* EXF-6108 cultivated on citrus waste. Melanin incorporation enhanced UV-shielding performance in SCP-based films containing 20% melanin which exhibited markedly reduced UVC/UVB transmittance and increased opacity, while reducing water vapor permeability in all film formulations. Tensile strength of BC-based films produced from glucose and cheese whey was increased from 4.4 ± 0.9 to 6.0 ± 1.3 MPa and from 3.7 ± 1.7 to 8.5 ± 2.4 MPa, respectively, following melanin incorporation. Overall, the findings demonstrate that waste-derived microbial materials enable sustainable packaging and promote a circular bioeconomy.

## 1. Introduction

The increasing accumulation of plastic waste and the generation of large quantities of agro-industrial residues have intensified the need for sustainable resource management and circular bioeconomy strategies. Packaging applications account for approximately 40% of global plastic production, with food packaging representing the largest end-use sector [1], thereby contributing substantially to environmental pollution and waste generation. Consequently, the replacement of petroleum-based plastics with biodegradable and renewable alternatives has become a major priority for both academia and industry.

Among the emerging bio-based materials, the ones of microbial origin such as single-cell protein (SCP) and bacterial cellulose (BC) have attracted considerable attention as sustainable alternatives for food-packaging applications because they can be produced through microbial fermentation without competing for agricultural land while exhibiting desirable film-forming properties [2,3,4]. Recent studies have demonstrated the potential of cheese whey-derived SCP as a novel edible packaging material, owing to its favorable film-forming ability, high nutritional value, satisfactory oxygen barrier properties, and promising sensory characteristics [5,6,7]. Similarly, BC has emerged as an attractive packaging biopolymer due to its unique three-dimensional nanofibrillar network, which provides high tensile strength, a high degree of polymerization and crystallinity, excellent water-holding capacity, mechanical stability and excellent biodegradability [8,9]. In addition, the abundant hydroxyl groups on the BC surface enable its functionalization through various modification strategies, allowing the enhancement of its physicochemical, barrier, and antimicrobial properties [10]. Collectively, SCP and BC represent two distinct complementary microbial material platforms, differing in their origins, structures, and functionalities, while sharing a common foundation in the biotechnological valorization of waste-derived resources with considerable potential for the development of next-generation sustainable food-packaging materials.

Meanwhile, cheese whey is among the most abundant agro-industrial residues, with approximately 9 L generated per kilogram of cheese produced [11]. Due to its high organic load, it also represents one of the most environmentally problematic dairy waste streams, necessitating effective management strategies [12]. A plethora of studies have demonstrated that the biotechnological valorization of cheese whey as a low-cost substrate for the production of both SCP [13,14] and BC [15,16] is highly promising. Therefore, converting cheese whey into microbial bioproducts such as BC and SCP simultaneously address waste management challenges while promoting sustainable material and protein production.

Melanin is a naturally occurring biopolymer found in animals, plants, fungi, yeasts, and bacteria, where it performs various protective biological functions. Owing to its unique physicochemical properties, including strong ultraviolet (UV) absorption, antioxidant activity, free radical scavenging ability, metal-chelating capacity, and protection against environmental stresses, melanin has attracted increasing attention as a sustainable functional biomaterial with applications in biomedicine, cosmetics, agriculture, textiles, and food-related technologies [17]. Microbial production has emerged as an attractive alternative to conventional pigment sources because it is independent of seasonal variations, allows controlled production, and can be integrated with the valorization of agro-industrial residues, supporting circular bioeconomy strategies [18]. MM can be biosynthesized through two main precursor routes, namely the DOPA and DHN pathways. Its formation is catalyzed by oxidative enzymes, including polyphenol oxidases, tyrosinases, laccases, and catechol oxidases. Copper ions play a critical role in the activity of these enzymes by facilitating molecular oxygen coordination and the proper orientation of the reducing substrate during catalysis. Depending on the availability and accumulation of hydroxylated aromatic intermediates, which may result from enzymatic imbalances during metabolism, different melanin forms, such as eumelanin and pheomelanin, can be generated [17].

Melanin production is highly dependent on both cultivation conditions and the metabolic characteristics of the producing microorganism. Factors such as pH, growth-medium composition, and the use of either conventional substrates or waste-derived by-products can significantly influence biosynthetic efficiency and final yields [17,19]. Furthermore, metabolic engineering approaches, including the targeted modification or deletion of genes involved in competing pathways, can redirect metabolic flux toward melanin biosynthesis and thereby enhance production [19]. Importantly, productivity also varies substantially according to the microbial species and the substrate employed. For example, a *Streptomyces* sp. cultivated using marine residues achieved a melanin yield of 39.4 g/L [20].

These multifunctional properties also make microbial melanin a promising additive for biodegradable food-packaging materials. Its UV-shielding and antioxidant properties can reduce light-induced deterioration and oxidative degradation of food products, while its incorporation into bio-based polymeric matrices may enhance the functional performance of packaging films without compromising their sustainability [21]. However, the application of waste-derived microbial melanin in microbial biopolymer-based packaging materials remains largely unexplored, highlighting the need for the development of such novel systems.

Therefore, the aim of this study was to develop and evaluate two complementary microbial-based packaging platforms, namely bacterial cellulose and single-cell protein films, produced through the valorization of cheese whey and functionalized with microbial melanin biosynthesized from orange processing waste. The physicochemical, optical, barrier, and mechanical properties of the developed films were investigated to assess their potential as sustainable food-packaging materials. To the best of our knowledge, this is the first study integrating these waste-derived microbial products into a unified waste-to-packaging strategy.

## 2. Materials and Methods

### 2.1. Raw Materials & Pre-Treatment

Citrus wastes (orange peels) were obtained from a local shop in Lemnos (Greece), dried at 80 °C and ground with a laboratory grinder to obtain a constant particle size of 10 mm. To obtain free sugars, peels were immersed in deionized water (80 °C, 60 min, 280 rpm) followed by centrifugation (9000 rpm, 4 °C, 10 min) and stored at −20 °C until further use. Concentrations of free sugars of orange peel waste extract (OPWE) were determined by DNS method [22] and reached ~33.0 g/L.

Cheese whey was obtained from a regional cheese manufacturer (Lemnos, Greece) and preserved at −20 ± 2 °C for subsequent use. DCW was generated through sterilization at 121 °C for 20 min, followed by centrifugation at 9000 rpm for 15 min at 4 °C using a Universal 320R centrifuge (Hettich, Tuttlingen, Germany), and filtration. DCW contained approximately ~37.0 g/L lactose and a pH value of ~6.7.

### 2.2. Microbial Strains and Growth Media

Yeast strains *Exophiala phaeomuriformis* EXF-6108 and *Kluyveromyces marxianus* EXF-5288 were kindly provided by Infrastructural Centre Mycosmo, MRIC UL, Slovenia. Bacterial strain *Komagataeibacter rhaeticus* UNIWA AAK2 was kindly provided by the Department of Wine, School of Food Science, University of West Attica.

*E. phaeomuriformis* EXF-6108 and *K marxianus* EXF-5288 were maintained on YPDA slants, while *K. rhaeticus* was maintained on HS medium slants. All strains were stored at 4 °C and subcultured periodically.

Batch fermentations were carried out in 250 mL Erlenmeyer flasks with a 50 mL working volume. A 48 h preculture (T = 30 °C, 180 rpm) was used as the inoculum in all experiments, at 2% (*v*/*v*) for *E. phaeomuriformis* EXF-6108 and *K. marxianus* EXF-5288, and 10% (*v*/*v*) for *K. rhaeticus* UNIWA AAK2. The pH was controlled using 5 M NaOH for *E. phaeomuriformis* (pH 6) and *K. rhaeticus* (pH 6), and 5 M H_2_SO_4_ for *K. marxianus* (pH 3.5).

*E. phaeomuriformis* EXF-6108 was cultivated at 30 °C and 180 rpm using OPWE-derived sugars (20 g/L), supplemented with 5 g/L peptone, 5 g/L yeast extract, 1 g/L KH_2_PO_4_, 2 g/L MgSO_4_, and 1 g/L NaCl (C/N ≈ 6.7). *K. rhaeticus* UNIWA AAK2 was cultivated statically at 30 °C using deproteinized cheese whey (DCW), or glucose (≈20 g/L total sugars), supplemented with 5 g/L yeast extract, 5 g/L peptone, 2.7 g/L Na_2_HPO_4_, and 1.15 g/L citric acid. *K. marxianus* EXF-5288 was cultivated at 20 °C and 180 rpm using DCW as the sole carbon source (70 g/L lactose), with mineral salts (g/L): KH_2_PO_4_, 7.0; Na_2_HPO_4_, 2.5; MgSO_4_·7H_2_O, 1.5; FeCl_3_·6H_2_O, 0.15; CaCl_2_·2H_2_O, 0.15; ZnSO_4_·7H_2_O, 0.02; and MnSO_4_·H_2_O, 0.06, while urea (0.22%, *w*/*w*) served as the nitrogen source, as previously reported [13].

### 2.3. Biomass and BC Production

Yeast biomass was recovered through three successive centrifugation cycles performed at 9000 rpm for 10 min at 4 °C using a Universal 320R-Hettich centrifuge (Tuttlingen, Germany). The resulting biomass was quantified gravimetrically by drying the collected material at approximately 85 °C until a constant mass was reached.

BC pellicles formed at the air–liquid interface were harvested, washed thoroughly with deionized water, and purified by treatment with 1 M NaOH at 80 °C for approximately 80 min to remove bacterial cells. The membranes were subsequently rinsed with deionized water until pH neutralization and then dried at 40 °C until constant weight.

### 2.4. Extraction and Recovery of Microbial Melanin

Microbial melanin was extracted according to Selvakumar et al. [23] with some modifications. Briefly, after centrifugation of the fermentation medium, the supernatant’s pH was adjusted to 12 with 1 M NaOH and incubated overnight at 70 °C and 300 rpm to promote melanin solubilization. Subsequently, the pH was lowered to 2 with 5 M H_2_SO_4_ to precipitate the pigment. The precipitated melanin was collected by centrifugation (9000 rpm, 4 °C, 10 min) and washed repeatedly with deionized water until neutral pH was achieved. To further purify the pigment, the alkaline dissolution–acid precipitation procedure was repeated twice. After each cycle, the precipitated melanin was collected by centrifugation and washed repeatedly with deionized water until neutral pH was achieved. After that, the purified microbial melanin was concentrated at 10 mg/mL and the resulting melanin stock solution was stored for further use.

### 2.5. Fourier-Transform Infrared Resonance (FT-IR) Analysis of Microbial Melanin

Fourier-transform infrared (FT-IR) spectroscopy was performed using a PerkinElmer Spectrum 100 IR spectrometer to characterize purified microbial melanin. Synthetic melanin (Sigma-Aldrich, St. Louis, MI, USA) was used as a reference standard. Samples were analyzed under a nitrogen atmosphere over the spectral range of 380–4000 cm^−1^ with a resolution of 4 cm^−1^. Two spectra were acquired for each sample at different loadings to ensure reproducibility. The purified microbial melanin was identified by comparison with the FT-IR spectrum of the synthetic melanin standard.

### 2.6. Development of SCP- and BC-Based Films Enriched with MM

#### 2.6.1. Development of SCP-Based Films Enriched with MM

SCP-based films were prepared according to Koukoumaki et al. [5] with minor modifications. Briefly, dry biomass (7%, *w*/*w*) was dispersed in deionized water, and the pH was adjusted to 8.0 with 0.5 M NaOH. Melanin stock solution was incorporated at final concentrations of 10 or 20% (*v*/*v*), after which the film-forming mixture was heated at 80 °C for 30 min to induce protein denaturation. The mixture was then rapidly cooled, and 50% (*w*/*w*, based on dry biomass) glycerol was added as a plasticizer. After homogenization (5500 rpm, 15 min; HG-15D, Witeg, Wertheim am Main, Germany), the solution was degassed in an ultrasonic bath (P70H, Elma Ultrasonic, Weinfelden, Switzerland) for 20 min.

The degassed film-forming solution was cast onto 9 cm Petri dishes and dried in an environmental chamber at 25 ± 1 °C and 55 ± 2% relative humidity for 24–48 h. The dried films were carefully peeled from the casting surface and conditioned at 25 ± 1 °C before further characterization. A control batch was prepared without melanin addition into solution.

#### 2.6.2. Development of BC-Based Films Enriched with MM

Wet BC pellicles were submerged in melanin stock solution for 48 h at room temperature to allow absorption. Subsequently, BC was rinsed with deionized water to remove any excess solution and neutralize the pH and were dried at 40 °C until constant weight. BC pellicles without melanin addition served as control. The melanin loading was standardized by using the same melanin stock solution concentration (10 mg/mL), immersion time (48 h), temperature, rinsing, and drying conditions for all BC pellicles. The actual amount of melanin retained by the BC matrix was not quantitatively determined.

### 2.7. Characterization of SCP- and BC-Based Films Enriched with MM

#### 2.7.1. Film Thickness

Film thickness was determined using a digital thickness gauge (F.F. Group Tools, Frankfurt, Germany) with an accuracy of 0.002 mm. Measurements were taken at three different locations on each film prior to characterization, and the mean value was used for subsequent analyses. Film thicknesses were comparable among the different formulations, with no significant differences observed (Tables S1 and S2).

#### 2.7.2. Light Transmission and Film Opacity

The optical properties of the films were determined using a UV–Vis spectrophotometer (UV-1900i, Shimadzu, Kyoto, Japan). UV–Vis absorbance spectra were measured in the wavelength range of 200–800 nm, while film opacity was calculated from the absorbance at 600 nm using the following equation:

(1) Opacity = A600/x

where A600 is the absorbance at 600 nm and x is the film thickness (mm).

#### 2.7.3. Analysis of Color Parameters

Film color characteristics were assessed with a Lovibond LC100 Spectrocolorimeter (Dortmund, Germany)). The measurements included the CIELAB parameters L*, a*, and b*, along with hue angle (h*) and chroma (C*). The L* scale ranged from 0 (black) to 100 (white), whereas positive and negative a* values represented red and green tones, respectively. Similarly, positive b* values indicated yellow coloration, while negative values corresponded to blue tones.

#### 2.7.4. Moisture Content, Solubility and Swelling Index Analysis

Moisture content (MC%) of film stripes (2 × 2 cm) was determinate at 105 °C according to Koukoumaki et al. [5], as follows:

MC (%) = [(M_0_ − M_1_)/M_0_] × 100 (2)

where M_0_ refers to initial film weight and M_1_ refers to film weight after drying.

Solubility (S%) and Swelling Index (S.I %) were calculated according to Papadaki et al. [24], according to the following equations:

S (%) = [(S_0_ − S_1_)/S_0_] × 100 (3)

where S_0_ = the initial film weight (g) and S_1_ = weight of dried film (g).

S.I (%) = [(S.I_1_ − S.I_0_)/S.I_0_] × 100 (4)

where S.I_0_ = the initial film weight (g) and S.I_1_ = weight of dried film (g).

#### 2.7.5. Water Vapor Permeability (WVP)

Water vapor permeability (WVP) was determined using the gravimetric cup method based on ASTM, Procedure B (1995) [25], following the methodology reported by Ramos et al. [26] with minor modifications. Deionized water was placed inside permeability cups, which were subsequently sealed with the film samples. The assembled cups were maintained in an environmental chamber at 25 °C and a relative humidity of 50 ± 2% for 7 days.

The water vapor transmission rate (WVTR) was calculated from the slope of the linear regression of the weight gained over time in days (d) (R^2^ ≥ 0.99). WVP (g⋅mm/m^2^⋅d⋅kPa) was then calculated using the following equation:

WVP = WVTR × x/Δp(5)

where WVTR (g/m^2^ d) is the slope (g/d) divided by the transfer area of the film (m^2^), x (mm) is the film thickness, and Δp (kPa) is the partial water vapor pressure across the film.

#### 2.7.6. Mechanical Properties

Mechanical properties, including tensile strength (TS) and elongation at break (E), were determined following the procedures described in ASTM D882 [27] and Piccirilli et al. [28]. Measurements were performed using a Texture Analyzer (TA.XT.plus C, Stable Micro Systems, Surrey, UK) equipped with A/MTG Mini Tensile Grips, and the data were analyzed using Texture Exponent Software (Version 6.1.18.0, Stable Micro Systems).

Film specimens were cut into strips measuring 10 × 60 mm, with an initial grip separation of 30 mm. Priors to analysis, films were conditioned in a test chamber at 25 ± 1 °C for 24 h at a relative humidity (RH) of 55 ± 2%.

The tests were conducted at a crosshead speed of 0.05 mm/s to obtain stress–strain curves. Tensile strength (TS, MPa) and elongation at break (E, %) were calculated using the following equations:

(6) TS (MPa) = F/(a × x) × 10^−6^

(7) E (%) = (d/L) × 100

where F is the maximum force (N), a is the film thickness (m), x is the film width (m), d is the elongation at the point of rupture (mm), and l is the initial gauge length of the film specimen (mm).

Strain rate (s^−1^) was calculated according to the following equation:

ε = v/L(8)

where v is the crosshead speed (mm/s) and lis the initial gauge length of the film specimen (mm).

### 2.8. Statistical Analysis

All experiments in films were performed using three independent batches (*N* = 3). For each batch, every measurement was carried out in triplicate, and the mean value was used for subsequent statistical analysis. The resulting data were organized in Microsoft Excel and statistically evaluated using XLSTAT software (Version 2018.1, Addinsoft, Paris, France). Differences among treatments were assessed by one-way analysis of variance (ANOVA), followed by Tukey’s honestly significant difference (HSD) post hoc test for multiple comparisons. Statistical significance was established at *p* ≤ 0.05. Two independent fermentation batches were prepared for each experimental condition (*N* = 2). The results presented are expressed as the mean of the measurements obtained from these independent biological replicates.

## 3. Results

### 3.1. Fourier-Transform Infrared Resonance (FT-IR) Analysis of Microbial Melanin

FT-IR spectra of synthetic melanin and melanin extracted from *E. phaeomuriformis* displayed the characteristic absorption bands associated with melanin (Figure 1). A broad band at 3660–3014 cm^−1^ was assigned to the stretching vibrations of O–H groups from carboxylic acids and phenolic hydroxyls, as well as N–H stretching of aromatic amino groups within the indole and pyrrole structures of melanin. The absorption band at 2924 cm^−1^ corresponded to C–H stretching vibrations of aliphatic methylene groups, while the peak at 1710 cm^−1^ was attributed to the C=O stretching of carbonyl groups. The band at 1612 cm^−1^ was assigned to vibrations of carboxylic acid (COOH) groups. The absorption bands in the 1481–1373 cm^−1^ region were attributed to vibrations associated with the indolic structure of melanin, including C–N/C=N and conjugated aromatic vibrations, which are characteristic of the melanin polymer. The band at 759 cm^−1^ was assigned to the out-of-plane bending vibration of aromatic C–H bonds, a characteristic feature of melanin pigments [26].

Notably, both spectra exhibited the characteristic melanin peaks at 1511, 1385, and 759 cm^−1^, supporting the presence of melanin-like pigment in the pigment extracted from *E. phaeomuriformis*. The close agreement between the FT-IR spectra of the extracted and synthetic melanin indicates that the fungal pigment shares the principal structural features of the reference melanin.

### 3.2. Light Transmittance

The UV–Vis transmittance spectra of the BC- and SCP based films are presented in Figure 2 and Figure 3. Melanin incorporation affected the optical properties of the films in a polymer-dependent manner. In BC: Gly + MM films, the addition of melanin resulted in lower transmittance throughout most of the UV region (200–400 nm), indicating improved UV-shielding properties compared with the BC:Gly films. In contrast, only minor differences in UV transmittance were observed between BC:DCW films with and without melanin, suggesting a limited effect of melanin on the UV barrier properties of this film platform. Furthermore, increasing the microbial melanin concentration from 10% to 20% in SCP-based films markedly reduced UV transmittance, particularly in the UV-B (280–320 nm) and UV-A (320–400 nm) regions (Figure 3), demonstrating a concentration-dependent enhancement of the UV-blocking capacity. In the visible region (400–800 nm), melanin-containing SCP films exhibited lower light transmittance than the SCP: control films, indicating reduced transparency with increasing melanin content. This increase in opacity highlights a clear trade-off between UV shielding and material clarity; it provides excellent protection for light-sensitive food matrices, yet poses a limitation for packaging applications that mandate product visibility. The non-monotonic UV transmittance of the SCP: MM10 sample may arise from concentration-dependent differences in melanin dispersion, aggregation, and interactions with the SCP matrix, affecting both light absorption and scattering.

### 3.3. Color Analysis & Film Opacity of BC- and SCP-Based Films

As shown in Table 1, regarding BC-based films, both the change in the cultivation substrate and the incorporation of melanin resulted in significant differences in the color parameters (*p* < 0.05). Microbial melanin incorporation significantly decreased the L* values, indicating a darker film appearance, while simultaneously increasing film opacity. Specifically, L* values of BC films produced by glucose consumption decreased from 77.2 ± 0.5 to 37.4 ± 1.8 and from 84.1 ± 0.7 to 51.6 ± 3.0 for BC films cultivated in DCW.

A similar trend was observed for the SCP-based films (Table 2, Figure 4), where the increased melanin addition also significantly reduced L* values from 61.8 ± 2.0 (blank) to 43.0 ± 1.9 and 44.5 ± 2.4 (10% and 20% melanin addition respectively).

### 3.4. Water-Related Properties of BC- and SCP-Based Films

The cultivation substrate significantly affected the moisture content (MC) of the BC films. Films produced from glucose-grown bacterial cellulose exhibited a significantly higher moisture content (20.0 ± 1.6%) than those produced from DCW-grown bacterial cellulose (14.4 ± 1.9%). Melanin incorporation influenced the moisture content differently depending on the cultivation substrate. Specifically, the MC of BC films decreased significantly to 9.6 ± 0.7% following melanin incorporation, whereas a slight increase was observed in films produced from DCW-grown bacterial cellulose.

In contrast, melanin incorporation exerted a similar effect on the other water-related properties regardless of the cultivation substrate. Specifically, film solubility, swelling index, and water vapor permeability increased significantly in all melanin-containing films as shown in Table 3.

In SCP-based films, MM incorporation resulted in a slight decrease in moisture content, irrespective of the concentration of the additive. Film solubility also decreased significantly, from 48.3 ± 0.3% in the control films to 38.8 ± 0.9% and 36.8 ± 0.6% following the incorporation of 10% and 20% MM, respectively. In contrast, both the swelling index (SI) and water vapor permeability (WVP) increased progressively with increasing MM concentration, as presented in Table 4.

### 3.5. Mechanical Properties of BC- and SCP-Based Films

Melanin incorporation significantly affected the mechanical properties of the BC-based films. Specifically, the addition of melanin significantly increased both the tensile strength (TS) and elongation at break (E%) of the films, regardless of the cultivation substrate (glucose or DCW) (Figure 5a,b). The TS increased from 4.40 ± 1.13 to 6.04 ± 1.20 MPa for BC films and from 3.70 ± 1.21 to 8.54 ± 1.20 MPa for BC films. Similarly, the E% increased from 0.98 ± 0.21 to 1.33 ± 0.10% for BC films and from 1.71 ± 0.40 to 2.39 ± 0.10% for BC films.

In contrast, melanin incorporation adversely affected the mechanical properties of the SCP-based films. Tensile strength decreased progressively with increasing melanin concentration, from 1.24 ± 0.01 MPa in the control films to 0.41 ± 0.17 MPa in films containing 20% melanin (Figure 6a,b). Elongation at break showed a slight increase following the incorporation of 10% melanin, reaching 3.86 ± 0.0%; however, a further increase in melanin concentration to 20% resulted in a decrease in E%.

## 4. Discussion

Melanin incorporation significantly influenced the color characteristics of the developed films. Specifically, the L* value decreased in both SCP- and BC-based films, indicating reduced lightness and the formation of darker films. This behavior is consistent with previous studies on melanin-containing protein-based films [29] and cellulose-based films [30], which similarly reported a significant decrease in L* following melanin addition, reflecting the intrinsic dark pigmentation of melanin.

Melanin incorporation generally improved the UV shielding properties of the films, as demonstrated by the reduction in UV transmittance, particularly in the UV-B and UV-A regions. This behavior is aligned with previous studies that examined the incorporation of melanin in polymeric films [31,32] and is attributed to melanin’s aromatic structure, which enables efficient absorption of UV radiation and rapid conversion of photon energy from UV light into heat to prevent destructive photodegradation [33].

The moisture content (MC) of SCP-based films without melanin was 20.7 ± 0.3%, which is in agreement with previous findings for SCP-based films (25.9 ± 0.3%) reported by Koukoumaki et al. [5]. In the present study, melanin incorporation reduced the MC of both SCP- and BC-based films, with the exception of the BC:DCW samples, which exhibited a slight increase in MC. Similar observations have been reported for alginate/PVA films containing melanin nanoparticles [34]. However, several studies have shown that melanin incorporation generally increases the MC of films prepared from different biopolymers, including chitosan, alginate, and carrageenan [35,36,37]. These discrepancies are most likely related to the different physicochemical properties of the polymer matrices and the resulting interactions between melanin and the functional groups of each biopolymer. Such interactions may either decrease or increase the availability of free hydroxyl groups capable of binding water molecules, ultimately affecting the moisture content of the films.

A similar explanation can be proposed for the differences observed in water solubility. In the present study, melanin addition significantly decreased the solubility of SCP-based films, whereas it increased the solubility of BC-based films. The reduced solubility observed in SCP-based films may be attributed to the formation of intermolecular interactions between melanin and the polymer chains, resulting in a more compact polymer network and consequently limiting film dissolution in water, as previously suggested by [37]. Conversely, the increased solubility of BC-based films may indicate weaker interactions between melanin and the cellulose matrix or structural modifications that facilitated water penetration and polymer dissolution.

In contrast to the MC results, both film platforms exhibited increased water vapor permeability (WVP) and swelling index (SI) following melanin incorporation. A similar increase in swelling index after melanin addition has been reported for protein-based films [31]. Regarding WVP, most previous studies have demonstrated that melanin improves the water vapor barrier properties of biopolymer films, resulting in lower WVP values in cellulose-based films [32] as well as in gelatin-based films [29]. Nevertheless, increases in WVP have also been reported in cellulose-based films [38] and agar-based films [39], indicating that the effect of melanin is highly dependent on the polymer matrix and the quality of filler dispersion. An increase in WVP may result from non-uniform melanin distribution within the polymer network, leading to the formation of micro-voids or discontinuities that facilitate water vapor diffusion. Therefore, the increase in WVP observed in the present study is likely associated with the specific interactions between melanin and the SCP- and BC-based matrices, as well as possible structural heterogeneities introduced during film formation.

Regarding the effect of microbial melanin on the mechanical properties of BC-based films, the present study demonstrated a clear reinforcing effect regardless of the culture medium used for bacterial cellulose production. Specifically, the tensile strength increased from 4.4 ± 0.9 to 6.0 ± 1.3 MPa for BC films and from 3.7 ± 1.7 to 8.5 ± 2.4 MPa for BC films following melanin incorporation. Similar improvements have been reported for cellulose-based films containing melanin [32,40]. In contrast, melanin incorporation into SCP-based films resulted in a reduction in tensile strength, particularly at higher melanin concentrations. A similar decrease in mechanical strength has also been reported for other protein-based films containing melanin [41], suggesting that the reinforcing effect of melanin is not universal but strongly depends on the characteristics of the polymer matrix.

The opposite mechanical responses observed for BC- and SCP-based films are likely attributed to differences in the interactions between melanin and each polymer matrix. In cellulose-based films, the abundance of hydroxyl groups may promote intermolecular interactions with the functional groups of melanin, potentially including hydrogen bonding. In case of BC-based films, the simultaneous increase in tensile strength and decrease in WVP observed following melanin incorporation are consistent with the formation of a more cohesive BC network, which may facilitate stress transfer, increased tensile strength, and reduced chain mobility, which is reflected by lower elongation at break. Similar reinforcement mechanisms have been reported for melanin-containing cellulose and PVA nanocomposites, where homogeneous melanin dispersion and strong interfacial interactions enhanced the mechanical performance [42].

On the contrary, in protein-based matrices, melanin may not act as an efficient reinforcing agent. Lower polymer–filler compatibility and disruption of existing protein–protein interactions may reduce the continuity of the polymer network and create stress-concentration sites, ultimately decreasing tensile strength. Therefore, the effect of melanin on the mechanical properties of biopolymer films depends primarily on polymer–melanin compatibility and the quality of melanin dispersion within the polymer matrix.

## 5. Conclusions

The present study demonstrates the feasibility of the holistic valorization of agro-industrial by-products, namely cheese whey and orange processing residues, for the development of alternative packaging materials following the biorefinery concept. Importantly, the comparison of BC- and SCP-based films revealed that the functionality of microbially produced melanin is strongly matrix-dependent, with the two polymer platforms exhibiting contrasting responses. In particular, melanin incorporation enhanced the mechanical properties of BC-based films, whereas it adversely affected the mechanical performance of SCP-based films. In contrast, its effect on UV-shielding properties varied between the two matrices and film formulations, with a pronounced concentration-dependent improvement observed in SCP-based films. The results indicate that microbially produced melanin is not a universal performance-enhancing additive, but rather a multifunctional bio-based component whose functionality is governed by the polymer matrix and polymer–melanin interactions. Thus, optimization of the matrix–melanin combination and melanin concentration is essential according to the targeted packaging application. Further research should focus on evaluating the performance of these material platforms in real food packaging systems, including shelf-life studies, migration and safety assessments, process scalability, and end-of-life biodegradation or recyclability, to facilitate their industrial implementation within a circular bioeconomy framework.

## Figures and Tables

**Figure 1:**
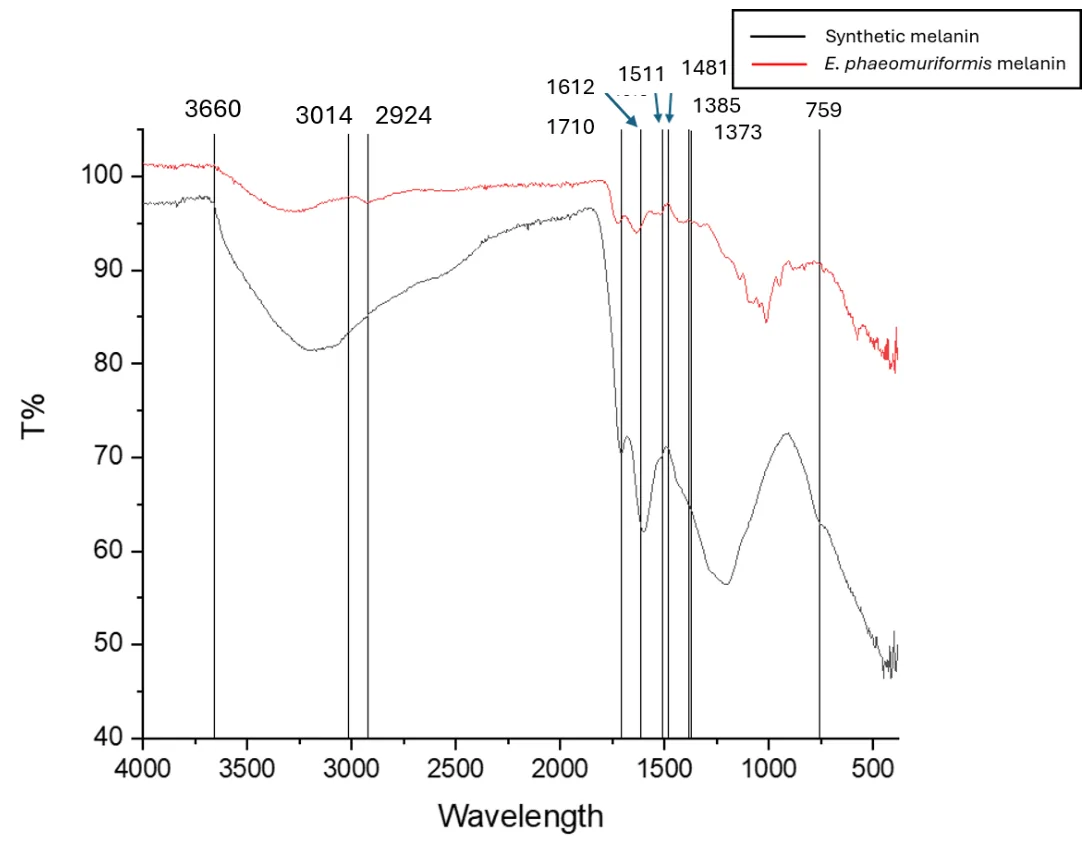
Fourier-transform infrared (FT-IR) spectra of synthetic melanin and melanin extracted from *Exophiala phaeomuriformis* strain EXF-6108 cultivated in OPWE.

**Figure 2:**
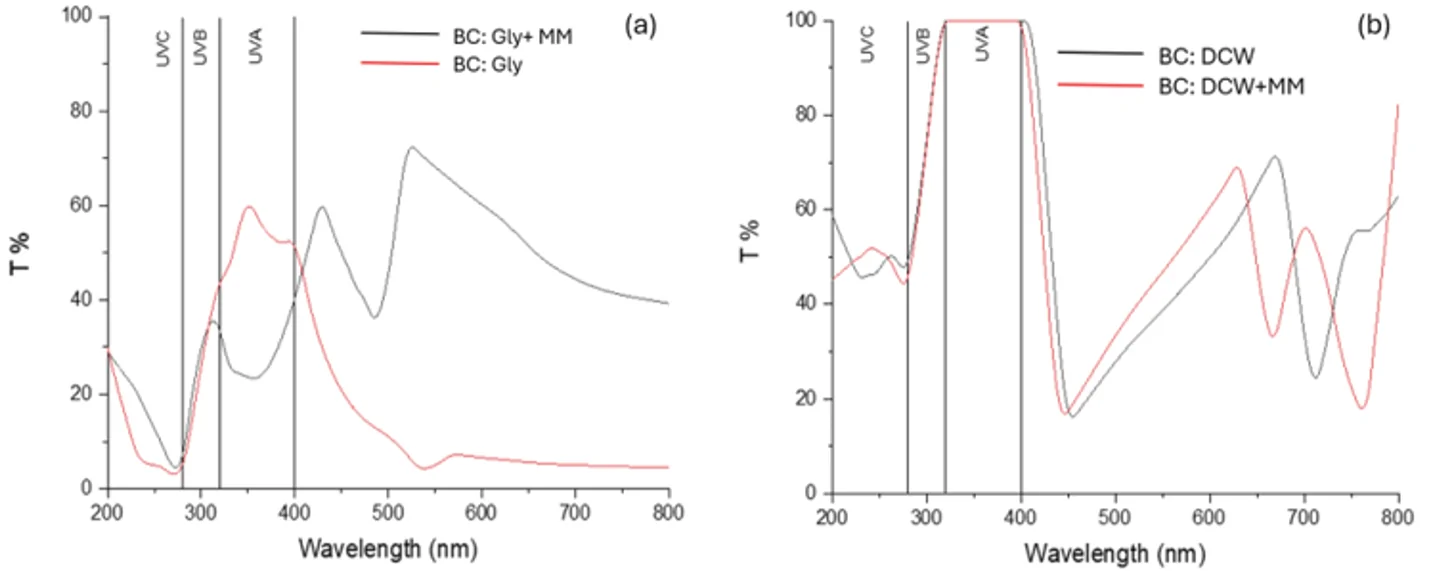
UV–Vis transmittance spectra of the BC-based films cultivated in glucose (**a**) and in deproteinized cheese whey (**b**), with and without microbial melanin addition.

**Figure 3:**
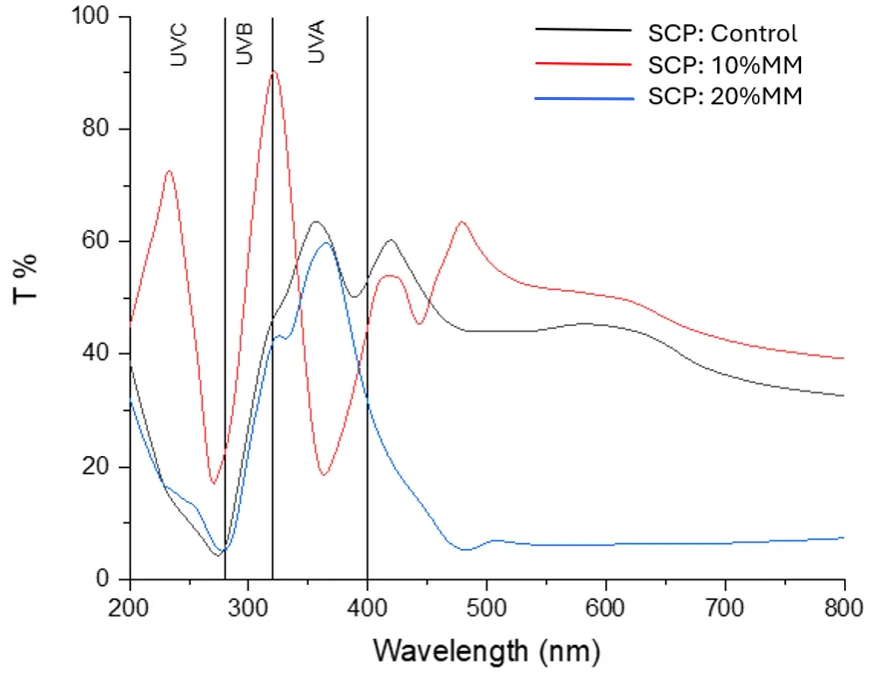
UV–Vis transmittance spectra of the SCP-based films with and without microbial melanin addition.

**Figure 4:**
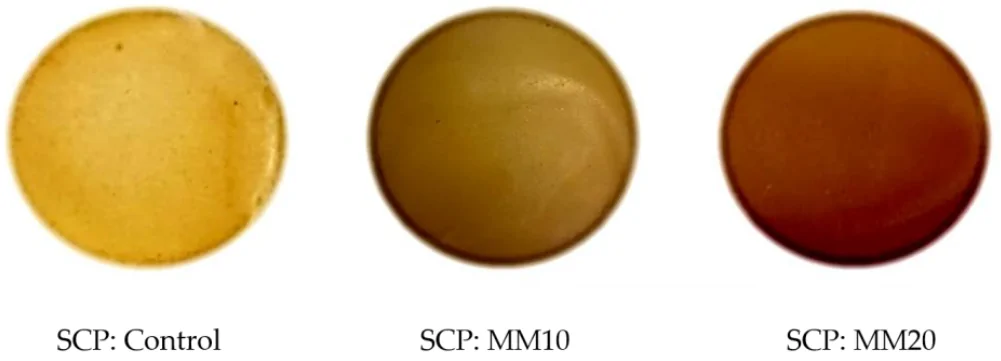
Developed SCP-based films with microbial melanin addition at 10 and 20% *v*/*v*.

**Figure 5:**
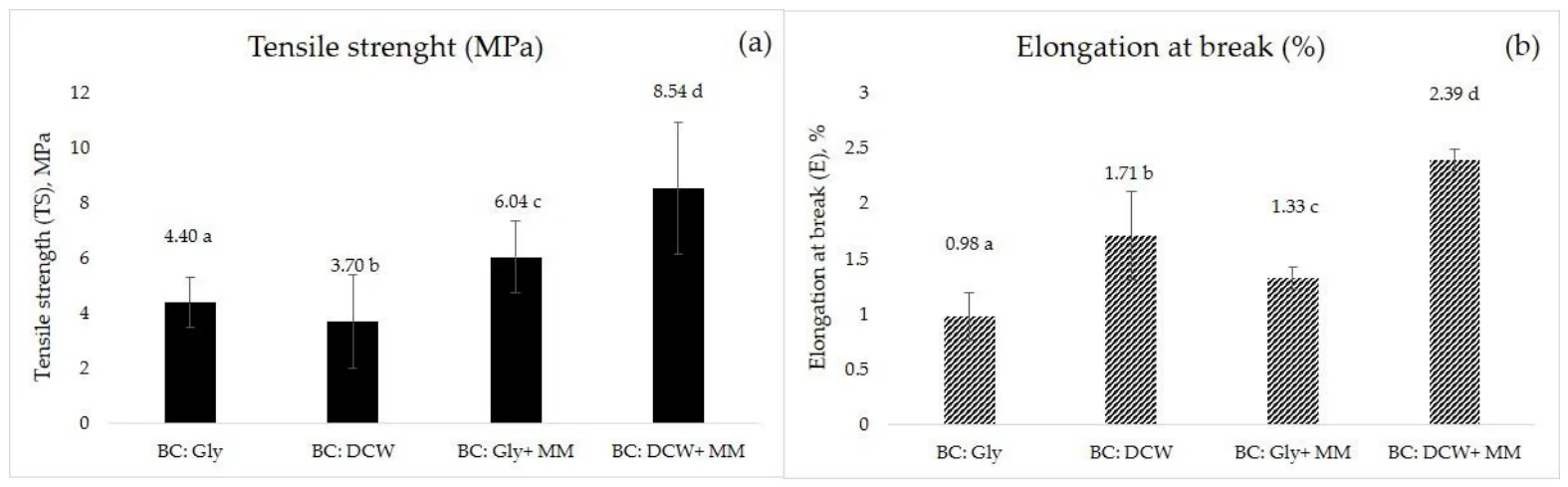
Mechanical properties in terms of tensile strength (MPa) (**a**) and elongation at break (%) (**b**) of BC-based films. Results are expressed as mean values accompanied by their corresponding standard deviations. Within each parameter, values marked with different lowercase superscript letters differ significantly (*p* < 0.05). All analyses were carried out using three independent experimental replicates (*N* = 3).

**Figure 6:**
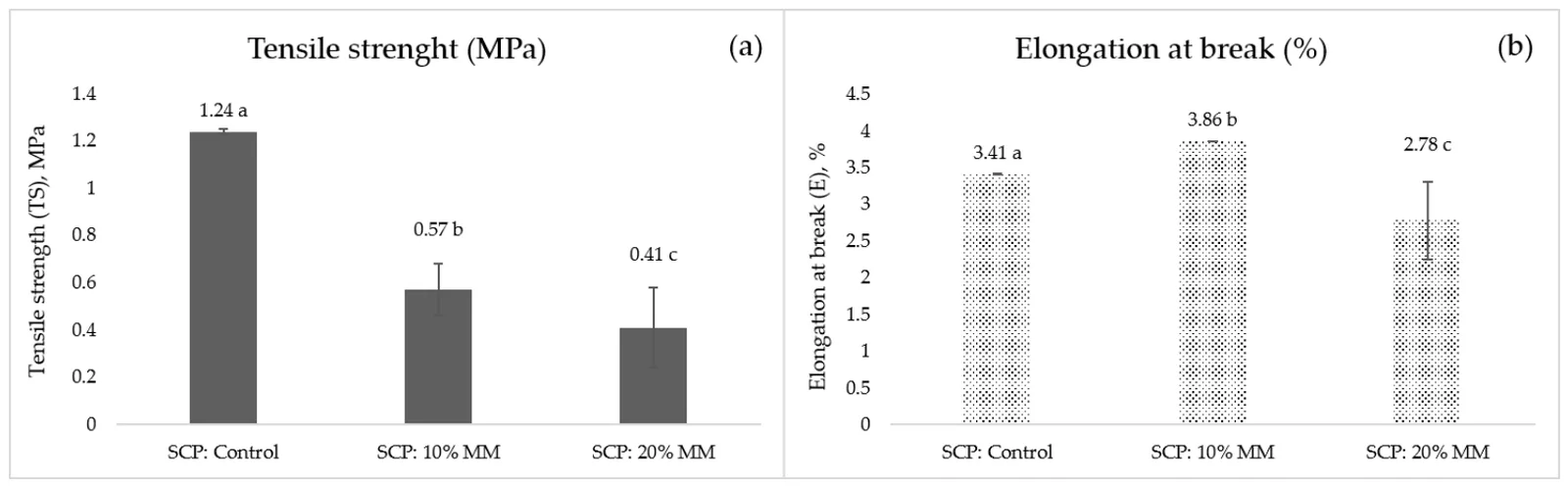
Mechanical properties in terms of tensile strength (MPa) (**a**) and elongation at break (%) (**b**) of SCP-based films. Results are expressed as mean values accompanied by their corresponding standard deviations. Within each parameter, values marked with different lowercase superscript letters differ significantly (*p* < 0.05). All analyses were carried out using three independent experimental replicates (*N* = 3).

**Table 1:** Optical properties of BC-based films with and without microbial melanin incorporation.

BC-Based Films	L*	a*	b*	C*	h*	Film Opacity
BC: Gly	77.2 ± 0.5 ^a^	4.4 ± 0.4 ^a^	16.4 ± 2.1 ^a^	17.0 ± 2.1 ^a^	74.9 ± 0.7 ^a^	0.8 ± 0.0 ^a^
BC: DCW	84.1 ± 0.7 ^b^	3.4 ± 0.1 ^b^	1.2 ± 0.7 ^b^	3.7 ± 0.3 ^b^	341.6 ± 7.9 ^b^	0.3 ± 0.0 ^b^
BC: Gly + MM	37.4 ± 1.8 ^c^	10.4 ± 0.4 ^c^	15.0 ± 1.8 ^c^	18.3 ± 1.7 ^c^	55.0 ± 2.3 ^c^	1.2 ± 0.0 ^c^
BC: DCW + MM	51.6 ± 3.0 ^d^	10.3 ± 0.8 ^d^	16.5 ± 1.7 ^d^	19.5 ± 1.8 ^d^	57.8 ± 0.9 ^d^	0.4 ± 0.0 ^d^

Results are expressed as mean values accompanied by their corresponding standard deviations. Within each parameter, values marked with different lowercase superscript letters differ significantly (*p* < 0.05). BC: bacterial cellulose; Gly: glucose; DCW: deproteinized cheese whey; MM: microbial melanin. All analyses were carried out using three independent experimental replicates (*N* = 3).

**Table 2:** Optical properties of SCP-based films with increased concentrations of microbial melanin.

SCP-Based Films	L*	a*	b*	C*	h*	Film Opacity
SCP: Control	61.8 ± 2.0 ^a^	11.3 ± 1.3 ^a^	42.7 ± 2.0 ^a^	44.2 ± 2.3 ^a^	75.2 ± 0.9 ^a^	0.1 ± 0.0 ^a^
SCP: MM10	43.0 ± 1.9 ^b^	21.4 ± 1.5 ^b^	41.5 ± 1.4 ^b^	46.8 ± 0.6 ^b^	62.7 ± 2.4 ^b^	0.2 ± 0.0 ^b^
SCP: MM20	44.5 ± 2.4 ^c^	21.1 ± 1.1 ^b^	41.0 ± 3.3 ^b^	46.2 ± 2.4 ^b^	62.6 ± 2.4 ^b^	1.1 ± 0.0 ^c^

Results are expressed as mean values accompanied by their corresponding standard deviations. Within each parameter, values marked with different lowercase superscript letters differ significantly (*p* < 0.05). SCP: single cell protein; MM10: 10% addition of microbial melanin; MM20: 20% addition of microbial melanin. All analyses were carried out using three independent experimental replicates (*N* = 3).

**Table 3:** The effect of microbial melanin of BC-based films in water-related properties.

BC-Based Films	MC (%)	S (%)	S.I (%)	WVP (g⋅mm/(m^2^⋅d⋅kPa)
BC: Gly	20 ± 1.6 ^a^	43.1 ± 4.1 ^a^	607.2 ± 6.7 ^a^	3.4 ± 1.0 ^a^
BC: DCW	14.4 ± 1.9 ^b^	12.5 ± 2.4 ^b^	262.2 ± 8.0 ^b^	0.9 ± 0.1 ^b^
BC: Gly + MM	9.6 ± 0.7 ^c^	53.3 ± 2.9 ^c^	680.0 ± 11.6 ^c^	8.0 ± 3.0 ^c^
BC: DCW + MM	18.2 ± 1.2 ^d^	56.0 ± 4.5 ^d^	491.5 ± 4.7 ^d^	1.8 ± 0.7 ^b^

Results are expressed as mean values accompanied by their corresponding standard deviations. Within each parameter, values marked with different lowercase superscript letters differ significantly (*p* < 0.05). BC: bacterial cellulose; Gly: glucose; DCW: deproteinized cheese whey; MM: microbial melanin. All analyses were carried out using three independent experimental replicates (*N* = 3).

**Table 4:** The effect of different concentrations of microbial melanin in SCP-based films in water-related properties.

SCP-Based Films	MC (%)	S (%)	S.I (%)	WVP (g⋅mm/(m^2^⋅d⋅kPa)
SCP: Control	20.7 ± 0.3 ^a^	48.3 ± 0.3 ^a^	5.2 ± 0.3 ^a^	8.1 ± 3.2 ^a^
SCP: MM10	18.7 ± 0.3 ^b^	38.8 ± 0.9 ^b^	7.1 ± 0.3 ^b^	9.2 ± 3.5 ^b^
SCP: MM20	18.4 ± 0.4 ^b^	36.8 ± 0.6 ^c^	9.0 ± 0.2 ^c^	9.5 ± 3.7 ^c^

Results are expressed as mean values accompanied by their corresponding standard deviations. Within each parameter, values marked with different lowercase superscript letters differ significantly (*p* < 0.05). SCP: single cell protein; MM10: 10% addition of microbial melanin; MM20: 20% addition of microbial melanin. All analyses were carried out using three independent experimental replicates (*N* = 3).

## Data Availability

The original contributions presented in this study are included in the article/Supplementary Materials. Further inquiries can be directed to the corresponding author.

## Supplementary Materials

The following supporting information can be downloaded at: https://www.mdpi.com/article/10.3390/foods15193394/s1, Table S1: Thickness measurements of BC-based films with and without microbial melanin incorporation; Table S2: Thickness measurements of SCP-based films with increased concentrations of microbial melanin.

## Abbreviations

The following abbreviations are used in this manuscript:

SCP	Single Cell Protein
BC	Bacterial Cellulose
MM	Microbial melanin
DCW	Deproteinized cheese whey
