# Supplementary material for: Engineering Two Sustainable Packaging Platforms from Agricultural Waste: Microbial Melanin-Enhanced Single-Cell Protein and Bacterial Cellulose Films
Source: Foods. 2026 Sep 23;15(19):3394. doi: 10.3390/foods15193394 (PMC13663949; doi:10.3390/foods15193394)
Supplement: Supplementary file 1 [file foods-15-03394-s001.zip › foods-4519254-supplementary.pdf]

Table S1. Thickness measurements of BC-based films with and without microbial melanin incorporation.

Samples	Thickness (mm)
BC: Gly	0.021± 0.002[a]
BC: DCW	0.022± 0.001[a]
BC: Gly+ MM	0.023± 0.001[a]
BC: DCW+ MM	0.023± 0.002[a]

Results are expressed as mean values accompanied by their corresponding standard deviations. Within each parameter, values marked with different lowercase superscript letters differ significantly ($p < 0.05$). BC: bacterial cellulose; Gly: glucose; DCW: deproteinized cheese whey; MM: microbial melanin. All analyses were carried out using three independent experimental replicates (N=3).

Table S2. Thickness measurements of SCP-based films with increased concentrations of microbial melanin.

Samples	Thickness (mm)
SCP: Control	0.11± 0.01[a]
SCP: MM10	0.13± 0.01[a]
SCP: MM20	0.12± 0.02[a]

Results are expressed as mean values accompanied by their corresponding standard deviations. Within each parameter, values marked with different lowercase superscript letters differ significantly ($p < 0.05$). SCP: single cell protein; MM10: 10% addition of microbial melanin; MM20: 20% addition of microbial melanin. All analyses were carried out using three independent experimental replicates (N=3).
